# Carbohydrate Intake in the Context of Exercise in People with Type 1 Diabetes

**DOI:** 10.3390/nu11123017

**Published:** 2019-12-10

**Authors:** Sam Scott, Patrick Kempf, Lia Bally, Christoph Stettler

**Affiliations:** 1 Department of Diabetes, Endocrinology, Nutritional Medicine and Metabolism, Inselspital, Bern University Hospital and University of Bern, 3010 Bern, Switzerland; sam-scott@live.co.uk (S.S.); patrick.kempf@insel.ch (P.K.); lia.bally@insel.ch (L.B.); 2 Team Novo Nordisk Professional Cycling Team, 2144 Hills Ave NW, Atlanta, GA 30318, USA

**Keywords:** type 1 diabetes, carbohydrates, insulin, exercise, glycogen, hypoglycemia, glucose, fructose, hormones, glycemic index

## Abstract

Although the benefits of regular exercise on cardiovascular risk factors are well established for people with type 1 diabetes (T1D), glycemic control remains a challenge during exercise. Carbohydrate consumption to fuel the exercise bout and/or for hypoglycemia prevention is an important cornerstone to maintain performance and avoid hypoglycemia. The main strategies pertinent to carbohydrate supplementation in the context of exercise cover three aspects: the amount of carbohydrates ingested (i.e., quantity in relation to demands to fuel exercise and avoid hypoglycemia), the timing of the intake (before, during and after the exercise, as well as circadian factors), and the quality of the carbohydrates (encompassing differing carbohydrate types, as well as the context within a meal and the associated macronutrients). The aim of this review is to comprehensively summarize the literature on carbohydrate intake in the context of exercise in people with T1D.

## 1. Introduction

People with type 1 diabetes (T1D) are recommended to engage in regular physical exercise for a variety of health and fitness reasons [1]. It has been shown that physical activity reduces cardiovascular risk, through improved modifiable risk factors such as hypertension or dyslipidemia, and has a beneficial impact on additional factors such as body composition and psychological wellbeing in patients with T1D [2,3]. However, the effects of exercise on glycemic control are complex, and, despite significant advances in insulins and supportive technology, exercise remains a challenge for this population [4,5,6,7]. Indeed, fear of hypoglycemia remains the most important barrier to exercise for many individuals living with T1D [4,6,8]. Current guidelines to prevent exercise-induced hypoglycemia suggest insulin dose adaptation and/or ingestion of additional carbohydrates in the context of the exercise bout [9,10,11]. While the authors acknowledge that insulin adaptation and carbohydrate consumption are intertwined when it comes to managing glycemia during exercise, this review will focus on the literature pertaining to carbohydrate intake.

Physical exercise is a complex metabolic stressor with many intra- and inter-individual variables influencing glycemic response. Therefore, for the individual with T1D, carbohydrate consumption to prevent hypoglycemia and/or to fuel the exercise bout is a challenge encompassing a variety of aspects (i.e., the amount of carbohydrates, the issue of timing, and the type of carbohydrate). The strategies used to manage glycemia during exercise require knowledge of pre-exercise blood glucose, the amount of “insulin on board”, and the expected blood glucose response depending on the type and volume of the planned exercise bout. To complicate matters, the type of carbohydrate, and the context in which it is consumed (e.g., with other macronutrients such as fats or proteins), can have an additional impact on glycemic response, and therefore insulin requirements, in the individual with T1D. Although the primary motivator for carbohydrate consumption before, during and after exercise for people with T1D is the avoidance of hypoglycemia, fuel provision for optimal performance, weight management and long-term glycemic control need to be considered. Additionally, from a psychological point of view, it is important that a level of food enjoyment is maintained, to limit the risk of eating disorders or food fixation, of which people with T1D are at greater risk than the general population [12,13].

The aim of this review is to summarize the literature, focusing on three central aspects, namely quantity, timing and type of carbohydrate intake, in the context of exercise in people with T1D. We will also discuss the impact of additional factors on glycemia and how the type of carbohydrate may be manipulated to manage blood glucose during and after exercise in people with T1D.

## 2. Physiology of Exercise in Type 1 Diabetes

Blood glucose responses to exercise vary considerably both between and within individuals with T1D, depending on numerous factors including (but not limited to) the type, duration and intensity of exercise; level of circulating exogenous insulin during and after exercise; and pre-exercise blood glucose concentration. Exercise is generally classified as ‘aerobic’ (longer activities of moderate intensity) or ‘anaerobic’ (short, very intense activities), depending on intensity as well as the predominant energy systems used, although many forms of exercise use a combination of the two. It is important that the reader understands how hormonal responses differ with the intensity of the exercise bout and the consequential effect on carbohydrate requirements for the individual with T1D.

In healthy individuals without T1D, tight control mechanisms exist so that glucose uptake into peripheral tissues is precisely matched by the rate of hepatic glucose production to maintain euglycemia [14]. During a bout of moderate-intensity aerobic exercise (50–80% $\dot{V}$O_2max_) in individuals without T1D, several counter-regulatory mechanisms are activated in a stepwise and hierarchical fashion to maintain euglycemia [15,16]. First, endogenous insulin secretion from the β-cells is suppressed to below fasting levels via sympathetic innervation of the islets of Langerhans [15]. The reduced insulin concentration enables the secretion of glucagon from the pancreatic *α*-cells into the portal vein, which stimulates hepatic glucose output to match the rate of glucose uptake into the skeletal muscles [16]. The decrease in insulin also sensitizes the liver to glucagon, which causes a rapid rise in cyclic AMP to stimulate glycogenolysis and gluconeogenesis [17,18,19]. As the exercise bout progresses, other counter-regulatory hormones are released, including catecholamines, growth hormone, aldosterone and cortisol, which stimulate hepatic glucose production and adipose tissue lipolysis, as well as inhibiting skeletal muscle glucose uptake, in order to protect against hypoglycemia [20,21]. As exercise intensity increases above 60% of $\dot{V}$O_2max_, lipid oxidation decreases, particularly in untrained individuals, and there is increased reliance on carbohydrates for energy provision [22].

In people with T1D, the glucoregulatory response to moderate-intensity exercise is impaired, mainly because they do not secret physiological amounts of endogenous insulin. Even the use of the latest modern therapeutic approaches (e.g., ultrafast-acting insulin analogues, insulin pumps, smart algorithms, hybrid closed-loop systems) cannot match the precise physiological metabolic regulation in those without T1D [23,24]. As a consequence, circulating systemic insulin concentrations lead to relative hyperinsulinemia during and after exercise in people with T1D. This is compounded by a dysregulated *α*-cell response, leading to lower glucagon levels and, hence, reduced hepatic glucose production [25]. High circulating insulin concentrations and skeletal muscle contraction exert additive effects on GLUT-4 translocation, resulting in heightened peripheral glucose uptake and decline in glycemia [26]. Exercise-induced increases in muscle perfusion [27] further increase insulin-mediated glucose disposal and, consequently, induce a drop in glycemia. Additional metabolic effects of higher insulin concentrations include the suppression of adipose tissue lipolysis and, therefore, fat oxidation in the skeletal muscle [28]. The unfavorable combination of changes in fuel selection and oxidation alongside the imbalance between peripheral glucose disposal and hepatic glucose production eventually results in an increased risk of hypoglycemia.

While mild- to moderate-intensity exercise generally increases the risk of hypoglycemia, intense exercise can have inverse effects [29]. The stimulation of counter-regulatory hormones may increase blood glucose levels, potentially leading to hyperglycemia [30,31,32]. In these situations, individuals with T1D may opt to correct their blood glucose with an insulin bolus after exercise [31]; however, care must be taken to avoid overcorrecting, as this can lead to severe post-exercise or nocturnal hypoglycemia. Conversely, in the absence of insulin (e.g., where the individual has removed their insulin pump or when insulin delivery is blocked or skipped), any level of exercise can lead to hyperglycemia and ketone formation. Many forms of physical activity, such as sports or spontaneous play, consist of intermittent periods of both moderate-intensity activity and short bursts of high-intensity activity. Circuit-based exercise has been shown to lead to a reduced drop in glycemia compared to moderate-intensity aerobic exercise upon basal insulin suspension at the start of exercise [33]. These forms of activity may attenuate the drop and reduce dependency on exogenous carbohydrates to maintain euglycemia [16].

It is clear that the intensity and duration of the exercise bout has important effects on the glucose response in the individual with T1D and, therefore, the strategy to manage glycemia. Depending on the specification of the individual insulin therapy, both basal or pre-meal bolus adaptations are possible strategies to prevent hypoglycemia during moderate-intensity exercise [33,34,35,36]. Adaptation of basal insulin is inherently easier for patients using insulin pumps or hybrid closed-loop systems, who are able to set temporary changes to their basal insulin and/or temporary changes to the glycemic target adapted to the exercise bout. A number of studies [33,34,37], as well as personal clinical experience, show that adaptation of the user’s insulin pump may be an important intervention to reduce the risk of hypoglycemia in the context of exercise. However, the exact mode of adaptation may be difficult and the underlying pharmacodynamics and pharmacokinetic changes are not entirely understood [38,39]. Additionally, using closed-loop insulin delivery may be insufficient to prevent hypoglycemia during exercise, whereas combining closed-loop insulin with additional snacking of 15–30 g of carbohydrate before exercise prevented all cases of hypoglycemia in a recent study [40]. For patients using multiple daily injections with modern ultra-long-acting insulin analogues, adaptation of basal insulin may be even more difficult or utterly impossible. Therefore, while further discussion of insulin adjustment strategies in the exercise context is beyond the scope of this review, it can be stated that the adequate supplementation with carbohydrates may offer a complementary approach with the potential advantage of being more flexible.

## 3. Quantity of Carbohydrate Supplementation in the Context of Exercise in Type 1 Diabetes

There are two important factors regarding the quantity of carbohydrates required in the context of exercise to consider: glycemic management and fueling the exercise bout. In essence, the quantity of recommended carbohydrates before, during and after a bout will depend on the blood glucose concentration, the type and intensity of exercise being performed, and the level of circulating insulin [9]. People with T1D are recommended to aim to start the bout of exercise with a stable blood glucose concentration between 7 and 14 mmol/L when ketone levels are low in blood (i.e., <1.5 mmol/L) or free/trace urine [9,11]. Before starting exercise, the individual with T1D is recommended to ingest 10–20 g glucose if glucose levels are <5.0 mmol/L and 10 g of carbohydrate if between 5.0 and 6.9 mmol/L [9].

The latest consensus guideline paper [9] contains a table summarizing the recommended quantity of carbohydrates to consume during exercise, depending on the duration of the exercise bout, blood glucose value and the amount of insulin on board (high or low). During a bout of moderate-intensity exercise lasting up to 30 min, in states of stable euglycemia, only a relatively small amount (10–20 g/h) of carbohydrate may be needed [9]. With increasing duration and intensity, additional amounts of carbohydrate are recommended, with up to 75–90 g/h when exercising for over an hour [9]. Grimm, et al. [41] compared the effects of adjusting carbohydrate intake in the context of exercise with or without insulin adjustment on the risk of hypoglycemia. During this study, the authors developed a table to guide carbohydrate requirements, depending on exercise intensity and duration (Table 1). Their study showed that it was possible to prevent almost all hypoglycemia during exercise, provided adequate carbohydrate was consumed. With the advent of continuous glucose monitoring technology, researchers have been able to integrate additional information to help decision making. For example, Riddell and colleagues [42] suggested the use of an algorithm (decision tree) based on glucose level and rate of change according to data derived from continuous glucose monitors to estimate carbohydrate requirements to maintain stable glycemia during exercise. Their algorithm suggested that if glucose was below 7.0 mmol/L, action was required by the individual, whereby 16–20 g was ingested depending on the value and rate of change.

These studies highlight the importance of adjusting insulin dosage to avoid the need to consume additional carbohydrates at the upper range of Table 1 [41]. Depending on the energy consumed throughout exercise, such carbohydrate intake may induce unintended weight gain. When larger amounts of carbohydrate are required to fuel prolonged endurance exercise, the maximum carbohydrate gut absorption capacity (1.2–1.7 g/min for glucose [43]) will become a limiting factor. In long-duration and high-intensity exercise situations, this may not only lead to an increased probability of gastrointestinal side effects, but also to a relative energy deficit, with an associated risk of hypoglycemia in those with T1D. In these situations, additional forms of carbohydrates other than glucose, with differing a mode of uptake and kinetics, may be beneficial (see Section 5 for more details).

## 4. Timing of Carbohydrate Intake in the Context of Exercise

While the issue of quantity is certainly of central importance, adequate handling of carbohydrate ingestion additionally encompasses aspects of timing. This section will discuss factors relating to the timing of a meal containing carbohydrate in the context of an exercise bout, and also factors that can impact the timing of the exercise itself (e.g., circadian rhythm).

Even with the most rapid-acting insulins, insulin is still likely to be active during exercise if the time between the meal and exercise is less than 2–3 h [23]. For this reason, many people with T1D choose to exercise in the morning in the fasted state. Yamanouchi, et al. [44] showed that walking after breakfast significantly decreased glycemia compared to walking before breakfast. However, when wanting to perform exercise in the post-absorptive state, which is often the most practical, due to the busy schedules of many active people, it may be wise to consume additional carbohydrates to account for the insulin on board [41,45]. To examine the effects of pre-exercise carbohydrate timing, West, Stephens, Bain, Kilduff, Luzio, Still and Bracken [45] compared glycemia during exercise 30, 60, 90 and 120 min after ingestion of a meal containing 75 g carbohydrate with a reduced insulin dose. While the insulin concentrations were comparable between the conditions, the risk of hypoglycemia was lowest in the 30 min condition. These results suggested that people with T1D may experience a reduced risk of hypoglycemia and higher lipid oxidation rates during exercise if the pre-exercise rest period is restricted to 30 min following the ingestion of low glycemic index carbohydrates and reduced insulin dose.

While awareness of hypoglycemia during exercise is comparably high in patients as well as health care providers, the issue of post-exercise, late-onset hypoglycemia may sometimes not be attributed the adequate importance [46,47]. Depending on the intensity and duration of an exercise bout, the increased risk of hypoglycemia can last for more than 30 h [46]. There are two main mechanisms for the increased risk of late onset hypoglycemia following exercise. First, liver and muscle glycogen stores must be restored after the exercise bout via circulating plasma glucose. Secondly, increased insulin sensitivity may persist for hours and even days, according to the duration and intensity of the exercise performed. The increased insulin sensitivity and continued extraction of glucose from the circulation may be related to increased glycogen synthase activity to replenish glycogen stores [48,49]. Enhanced glucose transporter 4 (GLUT 4) translocation and muscle microvascular perfusion may also persist after exercise, and are important for the ongoing changes in glucose uptake [50,51]. Special care may be required to prevent post-exercise hypoglycemia following afternoon or evening exercise, because there is a greater risk of nocturnal hypoglycemia [47,52,53,54]. Glucose requirements following moderate-intensity exercise performed late in the day exhibit a biphasic pattern, with increases occurring both immediately post and 7–11 h post exercise [55].

Nocturnal hypoglycemia is often particularly challenging, with over 50% of severe hypoglycemia episodes occurring during sleep [56,57]. Of note, exercise itself [58], as well as sleep [59], have been shown to impair awareness to hypoglycemia by blunting counter-regulatory responses. The risk of nocturnal hypoglycemia following 45 min of moderate-intensity exercise performed in the afternoon has been suggested to be as high as 30–40% [47,53,54]. Nutritional strategies to reduce the risk of nocturnal hypoglycemia include ingestion of a bedtime snack [60]. A bedtime snack containing carbohydrate and protein is regularly suggested in clinical practice and frequently used by patients; however, the optimal composition of this snack is still unclear. Whole milk and slowly absorbed snacks such as chocolate and fruit have been shown to reduce the risk of late-onset hypoglycemia [61] (see Section 5 for more details).

### Influence of Circadian Variation in Glucose Kinetics and therefore Carbohydrate Requirements

The glycemic response to exercise and, therefore, carbohydrate requirements to prevent hypoglycemia can also be influenced by the time of day. For example, afternoon resistance exercise in people with T1D has been shown lead to declines in glycemia [62,63], whereas an almost identical resistance exercise protocol performed in the morning under fasting conditions resulted in either no change [64] or a mean increase [65] in blood glucose concentration. This was supported by a repeated measures design study comparing morning and afternoon resistance exercise, where the morning (fasting) exercise led to an increasing trend in blood glucose, while blood glucose declined during afternoon exercise [66]. There have been similar findings with fasted aerobic exercise. Ruegemer, et al. [67] observed declines in blood glucose following 30 min of aerobic exercise in the afternoon, with the same participants experiencing an increase in blood glucose when performed in the morning. Similarly, Scott, et al. [68] observed that participants performing both moderate aerobic exercise and high-intensity interval training (HIIT) in the fasted state did not experience declines in blood glucose during either exercise protocol. These findings contrast with the declines in blood glucose found during later day (fed state) aerobic exercise [33,54,62,69,70,71,72] and HIIT [33,54,69,70,71].

There are a number of possible explanations for the phenomenon observed with fasted exercise in the morning. The first is that lower circulating insulin during fasted exercise decreases the suppression of hepatic glycogenolysis, and consequently increases blood glucose during exercise. Secondly, counter-regulatory hormones such as growth hormone and cortisol are higher in the morning, resulting in a rise in blood glucose [73,74,75]. Although the theories behind the greater glucose stability in the morning remain unconfirmed, it can still be suggested that those struggling with hypoglycemia during exercise, and/or those trying to avoid additional carbohydrates to aid weight management, may have greater success with early morning/fasted exercise than they would with exercise later in the day.

## 5. Quality of the Ingested Carbohydrate and the Influence of other Macronutrients/Fiber

Carbohydrates come in various forms, which influence their metabolic fate and glycemic impact [76]. Monosaccharides, also called ‘simple sugars’, are the basic units of carbohydrate that cannot be hydrolyzed to simpler chemical compounds. Examples of monosaccharides include glucose, fructose and galactose, with glucose being the primary cellular fuel source in almost all human tissues. Monosaccharides are the building blocks of disaccharides (e.g., lactose, maltose, sucrose, isomaltulose) and polysaccharides (e.g., glycogen, cellulose, amylose, and amylopectin). The different characteristics of carbohydrates influence the rate of digestion, intestinal absorption and hepatic metabolism, which are key determinants of their glycemic impact and, hence, the rate of delivery to the skeletal muscle. Interestingly, depending on the type of carbohydrate, different and even partly insulin-independent metabolic pathways are used [77].

Although carbohydrates are the main macronutrient affecting glycemia, the consumption of a mixed meal containing varying quantities of fat, protein and fiber may further modify the glycemic response by influencing the absorption rate of carbohydrates, intestinal and pancreatic hormones (e.g., glucagon) and peripheral insulin sensitivity [78,79,80,81,82]. Various studies [78] showed that meals containing high amounts of protein or fat increased and delayed the glycemic response compared to a control meal with an equivalent carbohydrate content [78,83,84,85]. Thus, the ADA [86] guidelines suggest that people with T1D make insulin adjustments to compensate for the glycemic effects of high-protein or high-fat meals.

### 5.1. Glycemic Impact of Carbohydrates

The glycemic impact of carbohydrates is determined by two aspects. First, by its chemical structure and second, by other food components consumed simultaneously (e.g., fiber, protein/amino acids, fat). The glycemic response of carbohydrates is often quantified by the glycemic index (GI), which ranks them on a scale from 0 to 100 according to the extent to which they raise blood glucose [87]. Foods are considered to have a low GI with an index less than 55, mid GI if the index is 56–69 and high GI if more than 70. Glucose is assigned the maximal GI of 100, which is why it is the most suitable carbohydrate for treating hypoglycemia. A handful studies have investigated the effects of different GI carbohydrates in people with T1D under resting conditions [88,89,90] and in the context of an exercise bout [91,92,93,94,95]. For example, one crossover study in eight individuals with T1D compared the ingestion of 75 g of either low GI carbohydrate isomaltulose (GI = 32) or dextrose (GI = 92) 2 h before a 45 min treadmill run [91]. The authors [91] found that blood glucose increased half as much in the isomaltulose trial compared to the dextrose condition during the rest period and remained 21% lower for 3 h of recovery after exercise. During the later stages of the exercise bout there was lower carbohydrate and greater lipid oxidation rate in the isomaltulose trial. The low GI carbohydrate improved the blood glucose response and supported the continued use of lipids compared to high GI dextrose.

Low GI snacks consumed before bedtime have also been shown to reduce the risk of nocturnal hypoglycemia following exercise, due to delayed and enduring glycemic effects and lower rapid insulin requirements [93]. Thus, based on the limited number of studies, low GI meals appear to be more effective at reducing the risk of hyperglycemia and optimizing fuel utilization throughout exercise, while offering protection from hypoglycemia for around 8 h post exercise. However, rapid-acting carbohydrates will always be an adequate choice to rapidly correct low blood glucose values.

### 5.2. Fructose and Other Alternatives to Glucose

There may be potential for other forms of carbohydrates, such as galactose and fructose, to provide alternatives to glucose for meeting energy requirements, independently of insulin, in people with T1D. These sugars are metabolized very differently: glucose is preferentially metabolized by tissues such as the skeletal muscle and brain [96,97], whereas fructose and galactose are primarily metabolized in the liver [98,99,100]. Although there is currently no data on galactose and T1D, the results from studies using fructose as a means to attenuate the drop in glycemia during exercise are promising [101,102].

Fructose is a monosaccharide with the same molecular formula as glucose (C_6_H_12_O_6_) but a different structure: the main difference being the arrangement of –OH and =O groups, resulting in a ketogroup at position C2 in fructose. For this reason, its metabolism differs markedly compared to glucose. Fructose is primarily absorbed across the apical membrane of the intestinal enterocytes by glucose transporter 5 (GLUT5), whereas glucose is absorbed by sodium–glucose cotransporter 1 (SGLT1) [103]. After intestinal absorption, fructose is first metabolized in the splanchnic area (intestine and liver) whereas glucose passes directly into the systemic circulation [98]. Therefore, following fructose ingestion, plasma fructose concentration remains low (<0.5 mmol/L) [104], supporting its low GI of 18. As there is no physiological feedback on fructokinase, all intracellular fructose is converted to triose-phosphate, which is then metabolized to pyruvate, lactate, glucose, glycogen and fatty acids [104,105].

**Figure 1:**
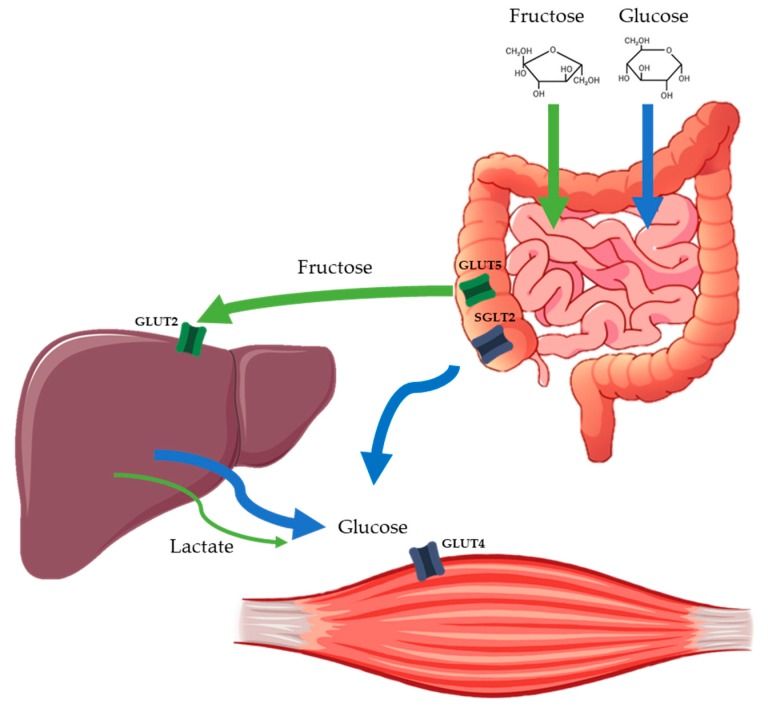
Simplified schematic of the main pathways involved in intestinal and hepatic fructose and glucose absorption.Orally ingested fructose is absorbed via different intestinal transport proteins to glucose and then almost completely extracted by the liver, where it is metabolized. In the liver, fructose is converted to primary energy substrates, such as lactate, glucose and lipids, which can be released into the circulation or stored in the liver as glycogen. This allows a supply of energy to maintain glycemia without the need for insulin. SGLT2 = sodium–glucose cotransporter 2; GLUT = glucose transporter protein.

Pre-exercise fructose intake may provide particular metabolic benefits to exercise and T1D, due to its attenuated glycemic effect and partial conversion to lactate and lipids (Figure 1) which can be used as alternative energy substrates by the muscle. The steady supply of slow release glucose from the liver following fructose ingestion ensures a more enduring glycemic effect. This may contribute towards maintaining glycemic stability during exercise while optimizing fat utilization [106]. In a study evaluating glucose–fructose co-ingestion compared to glucose alone at our institution, this metabolic trend of higher fat oxidation was confirmed [101]. Furthermore, unpublished data from our group has shown that ingestion of 20 g of fructose 30 min before an hour of cycling at 50% $\dot{V}$O_2max_ reduced the risk of hypoglycemia compared to water alone in patients using ultra-long acting insulin analogues who were, therefore, unable to adjust the basal insulin dose [102]. These data suggest that fructose may offer a simple alternative strategy to optimize glucose management during exercise in people with T1D, translating in higher fat oxidation and glycogen sparing, as well as a lower risk of hypoglycemia. Future research is needed to directly compare fructose and glucose, the effects of different exercise modalities, and the time of day that the exercise is performed.

As a cautionary note, ingestion of large amounts of fructose alone as a single dose (>50 g, but as low as 25 g in some individuals) may cause gastrointestinal distress [107]. Therefore, this approach should be evaluated on an individual basis, considering the spacing of doses or combination with glucose [101]. It is also important to acknowledge the potential dangers of chronic high-fructose consumption in the context of hypercaloric feeding and sedentary behavior [108,109,110]. Chronically high fructose consumption in rodents leads to hepatic and extrahepatic insulin resistance, obesity, type 2 diabetes and high blood pressure [111]. The evidence is less convincing in humans, but has been shown to lead to dyslipidemia and to impair hepatic insulin sensitivity [112]. However, this evidence derives from sedentary individuals, while studies in athletes have shown contrasting results [99].

## 6. Specific Considerations for Athletes with Type 1 Diabetes

### 6.1. Prolonged Endurance Exercise: Balancing Energy Requirements with Glycemic Control

It is important to acknowledge that a substantial number of people with T1D wish to greatly exceed the minimum activity guidelines, which means that some of the available studies provide limited use to these individuals and their healthcare providers. Within the literature, there are numerous examples of people with T1D undertaking impressive feats of endurance, lasting hours or even days, including ultramarathons, Ironman competitions, hiking over multiple days and long-distance cross-country skiing [113,114,115,116,117,118,119,120,121,122,123,124,125,126,127]. Such extreme feats of endurance would not be possible without close glucose monitoring and adjustment to food intake. Besides blood glucose management, there are multiple factors that the athlete with T1D must take into account to optimize performance and recovery during competition and key training sessions, including dehydration, electrolyte imbalances, glycogen depletion, gastrointestinal discomfort, and disturbances in acid-base balance [128].

Due to the complex inter-individual differences and the scarcity of research in this area, no standard recommendations concerning insulin adjustments and/or quantity of carbohydrate intake during prolonged endurance exercise exist for the athlete with T1D. In general, individuals with T1D wishing to engage in endurance exercise have to base their strategies on nutritional guidelines from studies conducted in people without T1D [128,129,130] and rely on past experience based on trial and error [125,131]. For example, our personal experience with professional cyclists with T1D showed that the riders consume a quantity of carbohydrates that is entirely in line with conventional recommendations during races (up to 90 g/h; see Table 2 for an overview of the carbohydrate quantities usually consumed by athletes in differing conditions). During prolonged endurance exercise, high carbohydrate intake is required to maintain the rate of carbohydrate oxidation necessary to sustain the exercise intensity and avoid glycogen depletion (muscle and liver), as this is a major cause of fatigue (reviewed by Hearris, et al. [132]). It is again important to emphasize that athletes with T1D need to balance their carbohydrate requirements with their workload, as well as simultaneously managing their blood glucose levels. One proposed strategy to combat this is to use a “reverse approach”, whereby a fixed amount of carbohydrate is consumed corresponding to the duration and intensity of the upcoming event, and insulin dose is individualized around this [126]. Adolfsson, Mattsson and Jendle [126] found that, when using such a strategy, glycemia was well maintained during a 90 km cross-country skiing race in a group of athletes with T1D. However, this reverse approach may not be possible for everyone and likely requires a lot of pre-planning, with factors such as competition stress or unexpected weather conditions likely to complicate matters.

A somewhat untouched topic in T1D research is the risk of relative energy deficiency in sport or RED-S. This refers to a situation where an athlete has insufficient energy intake relative to their training load, resulting in adverse health and impaired athletic performance [133,134]. Specific health consequences of RED-S include affected menstrual function, bone health, endocrine, metabolic, hematological, growth and development, psychological, cardiovascular, gastrointestinal, and immunological systems. Athletes at particular risk of RED-S are those involved in sports where high power-to-weight ratio is conducive to performance (e.g., cycling, triathlon, running), in weight category sports (e.g., boxing, lightweight rowing), and in aesthetic sports (e.g., dancing, gymnastics). The complexities of balancing glycemia, energy requirements for training, and long-term weight management may put athletes with T1D at particular risk of RED-S. It is important that the athletes, their coaches and their healthcare providers are aware of the potential risks of RED-S. Factors relating to RED-S in athletes with T1D, including training load, calorie intake and ensuring adequate recovery time alongside glycemic management, are complex and should be the focus of future research.

### 6.2. The Impact of Competition Stress on Carbohydrate Requirements

In athletes with T1D, additional factors related to glycemia and carbohydrate intake have to be considered. One aspect which may often be forgotten is the influence of competition stress [135]. This is where the release of stress hormones (glucagon, but also adrenaline and noradrenaline) before the start of an event [116] leads to a substantial increase in glucose levels. This may result in avoidance of carbohydrate intake at the start of a race, which, in turn, is associated with an increased risk of late onset hypoglycemia [136], since athletes may not be taking in enough fuel for the work required. Competition stress is a somewhat understudied area, as it is very difficult to reproduce in the laboratory and the effects are very individual. Therefore, a certain amount of trial and error with the guidance of a medical professional is required to work out a pre-race strategy that works for the individual.

### 6.3. Carbohydrate Loading

Carbohydrate loading is a common strategy used by athletes without T1D in the days leading up to a prolonged endurance event that is generally accepted to increase exercise performance and capacity in events lasting longer than 90 min [137]. A typical carbohydrate loading regimen is three days of increased carbohydrate intake between 8–12 g/kg/day, whereby carbohydrate contributes to 70–85% of the total energy intake [128]. In those without T1D, the increased carbohydrate intake is automatically matched by a greater endogenous insulin secretion to maintain glucose homeostasis. However, individuals with T1D need to adapt their insulin doses in accordance with the greater carbohydrate intake, which can be challenging [127,138]. There are very few studies investigating the benefits of and/or strategies to effectively carbo-load in individuals with T1D. Just over 20 years ago, McKewen, Rehrer, Cox and Mann [138] conducted a randomized crossover design in seven trained men with T1D to compare the effects of a 3-week moderate- vs. high-carbohydrate diet on glycogen stores, glycemic control and performance during a 15 min time trial. The high carbohydrate diet resulted in worse glucose control and performance during the time trial, leading the authors to conclude that a high carbohydrate diet prior to exercise is not beneficial, due to worse blood glucose control. More recently, Mattsson, Jendle and Adolfsson [127] investigated the effects of two days’ carbohydrate loading, followed by a high, intermittent carbohydrate intake during a prolonged 90 km cross country ski race in 10 people with T1D. In contrast to the findings of McKewen, Rehrer, Cox and Mann [138], Mattsson, Jendle and Adolfsson [127] reported good glycemic control during the two days of carbohydrate loading. Mattsson, Jendle and Adolfsson [127] suggest that this was due to the fact that they gradually increased the basal insulin doses during nights one and two of carbohydrate loading, and were careful to achieve stable glucose values before the start of the exercise bout. It is likely that they were also aided by the use of modern insulin preparations, as well as CGM technology, which was not available during the study by McKewen, Rehrer, Cox and Mann [138]. However, participants in the study by Mattsson, Jendle and Adolfsson [127] spent ~10% of their time in hypoglycemia, suggesting that adaptation of insulin was still challenging. In any case, individuals with T1D wishing to try carbohydrate loading before an athletic event may require more intensive blood glucose monitoring to avoid any deterioration of glycemia.

It was previously unknown whether people with T1D have poorer glycogen storage, which would make carbohydrate loading useless. Of note, previous ^13^C nuclear magnetic resonance spectroscopy (MRS) studies that measured hepatic glycogen under the physiologic conditions of mixed meal ingestion showed that poorly controlled individuals with T1D exhibit a defect of net liver glycogen synthesis that accumulates throughout the day and is most pronounced after the evening meal [139,140]. Furthermore, these authors observed a higher contribution of gluconeogenesis to glycogen synthesis in T1D (the so-called indirect glycogen synthesis pathway) compared to subjects without T1D. Intensification of insulin treatment normalized glycogen storage but not the contribution of gluconeogenesis to glycogen synthesis [140]. The latter finding supports the idea that even advanced insulin substitution regimens do not resemble the physiologic insulin secretion pattern, since the peripheral administration of insulin distorts the portal-to-peripheral insulin gradient, thereby affecting hepatic glycogen turnover [141]. Our group previously contrasted muscle and liver glycogen content in well-controlled individuals with T1D and matched controls without T1D using ^13^C-MRS and found no significant between group differences [142]. This suggests that, under adequate conditions, carbohydrate loading may benefit exercise performance and/or capacity in people with T1D.

## 7. Modifying/Additional Factors

### 7.1. Influence of Sex Hormones on Fuel Metabolism and Counter-Regulation

Within the T1D and exercise literature, the majority of published studies only include young healthy males, and those that have included females tend not to recognize the potential sex-related impact on their outcomes. As previously discussed [143,144], this is an important issue because, based on studies conducted in individuals without T1D, there are likely important sex-related differences in metabolic and neuroendocrine responses during exercise that will influence glycemia and, therefore, carbohydrate requirements. Sex hormones have important effects on the metabolic and neuroendocrine responses to exercise (reviewed by [145,146]) that are likely to influence carbohydrate requirements. In people without T1D, studies have shown clear sex-related differences in fuel metabolism during fasted exercise between men and premenopausal women [147,148,149,150]. During endurance exercise at the same relative intensity, females have a lower respiratory exchange ratio than men, indicative of a lower reliance on carbohydrate oxidation for energy provision during exercise [147,148,150]. In females, higher estradiol levels promote lipid oxidation and glycogen sparing, as well as greater sensitivity to the lipolytic action of catecholamines [151]. These metabolic differences between the sexes would suggest that men would have greater carbohydrate requirements around exercise than women. Interestingly, however, the sex-related differences in fuel selection during exercise are reduced when a carbohydrate load precedes the exercise bout [152]. This is of particular importance to people with T1D who regularly consume carbohydrate loads before and during exercise.

The female hormones, estrogen and progesterone, fluctuate predictably across the menstrual cycle in eumenorrhoeic women [153]. Women with T1D have important glycemic variability changes that are specific to the individual and linked to the phases of the menstrual cycle [154]. Women often wish to undertake physical activity or compete in sporting events at all stages of their menstrual cycle. It is therefore important to have an understanding of the hormonal influences on blood glucose and carbohydrate requirements. Studies have shown that the rate of appearance and disappearance of glucose during exercise is attenuated by therapeutic increases in circulating estrogen [155,156,157], or during the mid-luteal phase of the menstrual cycle, compared to the early follicular phase [148,158,159]. In females with T1D, there is an increased risk of hyperglycemia and decreased insulin sensitivity in the luteal phase compared to the follicular phase.

### 7.2. Eating Disorders or Anxiety related to Food

As discussed above, managing glycemia is complex and treatment goals can risk encouraging perfectionism, which, in turn, can lead to frustration due to the sheer improbability of obtaining glucose values in the target range 100% of the time. Certain aspects of T1D management increase the risk of developing eating disorders, particularly as emphasis is often placed on food selection and portion size. Compared to those without T1D, people living with T1D are at an increased risk of developing eating disorders [12,13,160,161] and other psychological disorders such as depression [162,163]. Weight management is a common motive for taking part in exercise [164]; however, carbohydrate requirements to avoid hypoglycemia during exercise can be high [165], which can be discouraging from a weight management point of view [8]. Of concern are previous reports that approximately 28% of female and 7% of male adolescents with T1D skip meals in an attempt to manage their weight [166]. Adolescents who engage in disturbed eating behaviors have poorer metabolic control [167], and insulin restriction is associated with increased risk of mortality [166]. Because of the risks, the latest ADA guidelines recommend that psychosocial care should be integrated as part of a patient-centered approach [86]. Future guidelines and strategies to improve glycemic control in the context of exercise should take these aspects into account, enabling people with T1D to have a positive attitude towards exercise, while allowing for a balanced approach towards nutrition and a healthy body image in this context.

## 8. Taking an Individualized Approach to Carbohydrate Intake

Practical use of the recommendations derived from the guidelines [9,10] can be difficult to follow due to the sheer number of inter- and intra-individual factors influencing glycemia during and after exercise [168]. Decision making can also be complicated because symptoms of hypoglycemia are often masked during exercise, which increases the risk of hypoglycemia if exercise continues [120]. The use of a customizable algorithm has been proposed [169,170] to help estimate carbohydrate requirements, and to improve the range of time and ease the burden of decision making. One example is the ECRES algorithm (Exercise Carbohydrate Requirement Estimating Software), which takes into account information regarding the individual’s usual insulin therapy (type, dose and schedule), usual carbohydrate intake and fitness level [171]. Based on this, the algorithm estimates plasma insulin concentration profile throughout the day using standard pharmacokinetic profiles, as well as taking into account insulin sensitivity. During the exercise bout, the algorithm uses expected average heart rate, exercise duration and actual glycemia to estimate carbohydrate requirements. Advances in artificial pancreas systems that link CGM to the user’s insulin pump to automatically adjust insulin levels, and therefore carbohydrate requirements, through intelligent algorithms, have the potential to ease the burden of exercise management in T1D. Integration of such hybrid-/closed-loop systems with automated exercise detection tools (e.g., heart rate monitors and/or activity monitors) may help to further reduce user input [172].

## 9. Conclusions

Physical exercise is a complex metabolic stressor with many intra- and inter-individual variables influencing glycemic response. Therefore, for the individual with T1D, carbohydrate consumption to prevent hypoglycemia and/or to fuel the exercise bout is a challenge encompassing a variety of aspects (i.e., the amount of carbohydrates, the timing in relation to an exercise bout, as well as the type of carbohydrate consumed). The strategies used to manage glycemia during exercise require knowledge of pre-exercise blood glucose concentration, the amount of “insulin on board”, and the expected blood glucose response, depending on the type and volume of the planned exercise bout. To complicate matters, the type of carbohydrate (e.g., the glycemic index) and context in which it is consumed (e.g., with other macronutrients such as fat or protein) can have an additional impact on the glycemic response and, therefore, insulin requirements, in the individual with T1D. Although the primary motivator for carbohydrate consumption before, during and after exercise for people with T1D is in the avoidance of hypoglycemia, fuel provision for optimal performance, weight management and long-term glycemic control need to be considered. Additionally, from a psychological point of view, it is important that a level of food enjoyment is maintained, to limit the risk of eating disorders or food fixation, of which people with T1D are at greater risk than the general population [12,13].

## Figures and Tables

**Table 1:** Amount of carbohydrates required to prevent hypoglycemia during exercises of different durations and intensities in people with type 1 diabetes. Adapted from Grimm, Ybarra, Berne, Muchnick and Golay [41].

Intensity	<20 min	20–60 min	>60 min
<60% HR_max_	0–10 g	10–20 g	15–35 g/h
60%–75% HR_max_	10–20 g	20–60 g	20–100 g/h
>75% HR_max_	20–30 g	30–100 g	30–150 g/h

HR_max_ = heart rate maximum.

**Table 2:** Guidelines for carbohydrate intake by endurance-trained athletes without type one diabetes (T1D), adapted from Burke [129], Jeukendrup [130] and the position of the Academy of Nutrition and Dietetics, Dietitians of Canada, and the American College of Sports Medicine guidelines [128].

Level of Activity	Carbohydrate Targets
Light (low intensity or skill-based activities)	3–5 g/kg bodyweight/day
Moderate (approximately 1 h per day)	5—7 g/kg bodyweight/day
High (e.g., 1–3 h moderate to high-intensity exercise)	6–10 g/kg bodyweight/day
Very high (e.g., >4/5 h of moderate to high-intensity exercise)	8–12 g/kg bodyweight/day
Extreme (e.g., elite cycle competition)	>12 g/kg bodyweight/day

Note: Nutrition goals for athletes are not static. Many athletes undertake a periodized program in which preparation for peak performance is achieved using different types of workout over the training calendar. Nutrition support needs to be periodized, taking the nutritional requirements of daily training sessions into account.

## References

[1] Colberg S.R., Sigal R.J., Yardley J.E., Riddell M.C., Dunstan D.W., Dempsey P.C., Horton E.S., Castorino K., Tate D.F. (2016). Physical Activity/Exercise and Diabetes: A Position Statement of the American Diabetes Association. Diabetes Care.

[2] Chimen M., Kennedy A., Nirantharakumar K., Pang T.T., Andrews R., Narendran P. (2012). What are the health benefits of physical activity in type 1 diabetes mellitus? A literature review. Diabetologia.

[3] Kennedy A., Nirantharakumar K., Chimen M., Pang T.T., Hemming K., Andrews R.C., Narendran P. (2013). Does exercise improve glycaemic control in type 1 diabetes? A systematic review and meta-analysis. PLoS ONE.

[4] Brazeau A.-S., Rabasa-Lhoret R., Strychar I., Mircescu H. (2008). Barriers to Physical Activity among Patients With Type 1 Diabetes. Diabetes Care.

[5] Kennedy A., Narendran P., Andrews R.C., Daley A., Greenfield S.M., Group E. (2018). Attitudes and barriers to exercise in adults with a recent diagnosis of type 1 diabetes: A qualitative study of participants in the Exercise for Type 1 Diabetes (EXTOD) study. Bmj Open.

[6] Lascar N., Kennedy A., Hancock B., Jenkins D., Andrews R.C., Greenfield S., Narendran P. (2014). Attitudes and barriers to exercise in adults with type 1 diabetes (T1DM) and how best to address them: A qualitative study. PLoS ONE.

[7] Quirk H., Blake H., Dee B., Glazebrook C. (2014). “You can’t just jump on a bike and go”: A qualitative study exploring parents’ perceptions of physical activity in children with type 1 diabetes. Bmc Pediatr..

[8] Scott S.N., Shepherd S.O., Andrews R.C., Narendran P., Purewal T.S., Kinnafick F., Cuthbertson D.J., Atkinson-Goulding S., Noon T., Wagenmakers A.J.M. (2019). A Multidisciplinary Evaluation of a Virtually Supervised Home-Based High-Intensity Interval Training Intervention in People with Type 1 Diabetes. Diabetes Care.

[9] Riddell M.C., Gallen I.W., Smart C.E., Taplin C.E., Adolfsson P., Lumb A.N., Kowalski A., Rabasa-Lhoret R., McCrimmon R.J., Hume C. (2017). Exercise management in type 1 diabetes: A consensus statement. lancet Diabetes Endocrinol..

[10] Adolfsson P., Riddell M.C., Taplin C.E., Davis E.A., Fournier P.A., Annan F., Scaramuzza A.E., Hasnani D., Hofer S.E. (2018). ISPAD Clinical Practice Consensus Guidelines 2018: Exercise in children and adolescents with diabetes. Pediatr. Diabetes.

[11] Narendran P., Jackson N., Daley A., Thompson D., Stokes K., Greenfield S., Charlton M., Curran M., Solomon T.P.J., Nouwen A. (2017). Exercise to preserve beta-cell function in recent-onset Type 1 diabetes mellitus (EXTOD)—A randomized controlled pilot trial. Diabet. Med. J. Br. Diabet. Assoc..

[12] Jones J.M., Lawson M.L., Daneman D., Olmsted M.P., Rodin G. (2000). Eating disorders in adolescent females with and without type 1 diabetes: Cross sectional study. Bmj.

[13] Wisting L., Skrivarhaug T., Dahl-Jorgensen K., Ro O. (2018). Prevalence of disturbed eating behavior and associated symptoms of anxiety and depression among adult males and females with type 1 diabetes. J. Eat. Disord..

[14] Gagliardino J.J. (2005). Physiological endocrine control of energy homeostasis and postprandial blood glucose levels. Eur. Rev. Med Pharm. Sci..

[15] Marliss E.B., Vranic M. (2002). Intense exercise has unique effects on both insulin release and its roles in glucoregulation: Implications for diabetes. Diabetes.

[16] Bally L., Zueger T., Buehler T., Dokumaci A.S., Speck C., Pasi N., Ciller C., Paganini D., Feller K., Loher H. (2016). Metabolic and hormonal response to intermittent high-intensity and continuous moderate intensity exercise in individuals with type 1 diabetes: A randomised crossover study. Diabetologia.

[17] Vranic M., Kawamori R., Pek S., Kovacevic N., Wrenshall G.A. (1976). The essentiality of insulin and the role of glucagon in regulating glucose utilization and production during strenuous exercise in dogs. J. Clin. Investig..

[18] Vranic M., Ross G., Doi K., Lickley L. (1976). The role of glucagon-insulin interactions in control of glucose turnover and its significance in diabetes. Metab. Clin. Exp..

[19] Zinker B.A., Mohr T., Kelly P., Namdaran K., Bracy D.P., Wasserman D.H. (1994). Exercise-induced fall in insulin: Mechanism of action at the liver and effects on muscle glucose metabolism. Am. J. Physiol..

[20] Chan O., Sherwin R. (2013). Influence of VMH fuel sensing on hypoglycemic responses. Trends Endocrinol. Metab. Tem.

[21] Donovan C.M., Watts A.G. (2014). Peripheral and central glucose sensing in hypoglycemic detection. Physiol. BethesdaMd.

[22] van Loon L.J., Greenhaff P.L., Constantin-Teodosiu D., Saris W.H., Wagenmakers A.J. (2001). The effects of increasing exercise intensity on muscle fuel utilisation in humans. J. Physiol..

[23] Heise T., Stender-Petersen K., Hovelmann U., Jacobsen J.B., Nosek L., Zijlstra E., Haahr H. (2017). Pharmacokinetic and Pharmacodynamic Properties of Faster-Acting Insulin Aspart versus Insulin Aspart Across a Clinically Relevant Dose Range in Subjects with Type 1 Diabetes Mellitus. Clin. Pharm..

[24] Brown S.A., Kovatchev B.P., Raghinaru D., Lum J.W., Buckingham B.A., Kudva Y.C., Laffel L.M., Levy C.J., Pinsker J.E., Wadwa R.P. (2019). Six-Month Randomized, Multicenter Trial of Closed-Loop Control in Type 1 Diabetes. N Engl J Med..

[25] Holst J.J., Holland W., Gromada J., Lee Y., Unger R.H., Yan H., Sloop K.W., Kieffer T.J., Damond N., Herrera P.L. (2017). Insulin and Glucagon: Partners for Life. Endocrinology.

[26] Camacho R.C., Galassetti P., Davis S.N., Wasserman D.H. (2005). Glucoregulation during and after exercise in health and insulin-dependent diabetes. Exerc. Sport Sci. Rev..

[27] Frank S., Jbaily A., Hinshaw L., Basu R., Basu A., Szeri A.J. (2018). Modeling the acute effects of exercise on insulin kinetics in type 1 diabetes. J. Pharm. Pharm..

[28] Saltiel A.R. (2016). Insulin Signaling in the Control of Glucose and Lipid Homeostasis. Handb. Exp. Pharmacol..

[29] Garcia-Garcia F., Kumareswaran K., Hovorka R., Hernando M.E. (2015). Quantifying the acute changes in glucose with exercise in type 1 diabetes: A systematic review and meta-analysis. Sports Med..

[30] Sigal R.J., Purdon C., Fisher S.J., Halter J.B., Vranic M., Marliss E.B. (1994). Hyperinsulinemia prevents prolonged hyperglycemia after intense exercise in insulin-dependent diabetic subjects. J. Clin. Endocrinol. Metab..

[31] Aronson R., Brown R.E., Li A., Riddell M.C. (2019). Optimal Insulin Correction Factor in Post-High-Intensity Exercise Hyperglycemia in Adults With Type 1 Diabetes: The FIT Study. Diabetes Care.

[32] Mitchell T.H., Abraham G., Schiffrin A., Leiter L.A., Marliss E.B. (1988). Hyperglycemia after intense exercise in IDDM subjects during continuous subcutaneous insulin infusion. Diabetes Care.

[33] Zaharieva D., Yavelberg L., Jamnik V., Cinar A., Turksoy K., Riddell M.C. (2017). The effects of basal insulin suspension at the start of exercise on blood glucose levels during continuous versus circuit-based exercise in individuals with type 1 diabetes on continuous subcutaneous insulin infusion. Diabetes Technol. Ther..

[34] Zaharieva D.P., McGaugh S., Pooni R., Vienneau T., Ly T., Riddell M.C. (2019). Improved open-loop glucose control with basal insulin reduction 90 minutes before aerobic exercise in patients with type 1 diabetes on continuous subcutaneous insulin infusion. Diabetes Care.

[35] Campbell M.D., Walker M., Trenell M.I., Luzio S., Dunseath G., Tuner D., Bracken R.M., Bain S.C., Russell M., Stevenson E.J. (2014). Metabolic implications when employing heavy pre- and post-exercise rapid-acting insulin reductions to prevent hypoglycaemia in type 1 diabetes patients: A randomised clinical trial. PLoS ONE.

[36] West D.J., Morton R.D., Bain S.C., Stephens J.W., Bracken R.M. (2010). Blood glucose responses to reductions in pre-exercise rapid-acting insulin for 24 h after running in individuals with type 1 diabetes. J. Sports Sci..

[37] Franc S., Daoudi A., Pochat A., Petit M.H., Randazzo C., Petit C., Duclos M., Penfornis A., Pussard E., Not D. (2015). Insulin-based strategies to prevent hypoglycaemia during and after exercise in adult patients with type 1 diabetes on pump therapy: The DIABRASPORT randomized study. DiabetesObes. Metab..

[38] McAuley S.A., Horsburgh J.C., Ward G.M., La Gerche A., Gooley J.L., Jenkins A.J., MacIsaac R.J., O’Neal D.N. (2016). Insulin pump basal adjustment for exercise in type 1 diabetes: A randomised crossover study. Diabetologia.

[39] Tagougui S., Taleb N., Rabasa-Lhoret R. (2018). The Benefits and Limits of Technological Advances in Glucose Management Around Physical Activity in Patients Type 1 Diabetes. Front. Endocrinol..

[40] Patel N.S., Van Name M.A., Cengiz E., Carria L.R., Tichy E.M., Weyman K., Weinzimer S.A., Tamborlane W.V., Sherr J.L. (2016). Mitigating Reductions in Glucose During Exercise on Closed-Loop Insulin Delivery: The Ex-Snacks Study. Diabetes Technol. Ther..

[41] Grimm J.J., Ybarra J., Berne C., Muchnick S., Golay A. (2004). A new table for prevention of hypoglycaemia during physical activity in type 1 diabetic patients. Diabetes Metab..

[42] Riddell M.C., Milliken J. (2011). Preventing exercise-induced hypoglycemia in type 1 diabetes using real-time continuous glucose monitoring and a new carbohydrate intake algorithm: An observational field study. Diabetes Technol. Ther..

[43] Jentjens R.L., Moseley L., Waring R.H., Harding L.K., Jeukendrup A.E. (2004). Oxidation of combined ingestion of glucose and fructose during exercise. J. Appl. Physiol..

[44] Yamanouchi K., Abe R., Takeda A., Atsumi Y., Shichiri M., Sato Y. (2002). The effect of walking before and after breakfast on blood glucose levels in patients with type 1 diabetes treated with intensive insulin therapy. Diabetes Res. Clin. Pract..

[45] West D.J., Stephens J.W., Bain S.C., Kilduff L.P., Luzio S., Still R., Bracken R.M. (2011). A combined insulin reduction and carbohydrate feeding strategy 30 min before running best preserves blood glucose concentration after exercise through improved fuel oxidation in type 1 diabetes mellitus. J. Sports Sci..

[46] MacDonald M.J. (1987). Postexercise late-onset hypoglycemia in insulin-dependent diabetic patients. Diabetes Care.

[47] Tsalikian E., Mauras N., Beck R.W., Tamborlane W.V., Janz K.F., Chase H.P., Wysocki T., Weinzimer S.A., Buckingham B.A., Kollman C. (2005). Impact of exercise on overnight glycemic control in children with type 1 diabetes mellitus. J. Pediatr..

[48] Bogardus C., Thuillez P., Ravussin E., Vasquez B., Narimiga M., Azhar S. (1983). Effect of muscle glycogen depletion on in vivo insulin action in man. J. Clin. Investig..

[49] Cartee G.D. (2015). Mechanisms for greater insulin-stimulated glucose uptake in normal and insulin-resistant skeletal muscle after acute exercise. Am. J. Physiol. Endocrinol. Metab..

[50] Wagenmakers A.J., Strauss J.A., Shepherd S.O., Keske M.A., Cocks M. (2016). Increased muscle blood supply and transendothelial nutrient and insulin transport induced by food intake and exercise: Effect of obesity and ageing. J. Physiol..

[51] Wagenmakers A.J., van Riel N.A., Frenneaux M.P., Stewart P.M. (2006). Integration of the metabolic and cardiovascular effects of exercise. Essays Biochem..

[52] Gomez A.M., Gomez C., Aschner P., Veloza A., Munoz O., Rubio C., Vallejo S. (2015). Effects of performing morning versus afternoon exercise on glycemic control and hypoglycemia frequency in type 1 diabetes patients on sensor-augmented insulin pump therapy. J. Diabetes Sci. Technol..

[53] Iscoe K.E., Campbell J.E., Jamnik V., Perkins B.A., Riddell M.C. (2006). Efficacy of continuous real-time blood glucose monitoring during and after prolonged high-intensity cycling exercise: Spinning with a continuous glucose monitoring system. Diabetes Technol. Ther..

[54] Maran A., Pavan P., Bonsembiante B., Brugin E., Ermolao A., Avogaro A., Zaccaria M. (2010). Continuous glucose monitoring reveals delayed nocturnal hypoglycemia after intermittent high-intensity exercise in nontrained patients with type 1 diabetes. Diabetes Technol. Ther..

[55] McMahon S.K., Ferreira L.D., Ratnam N., Davey R.J., Youngs L.M., Davis E.A., Fournier P.A., Jones T.W. (2007). Glucose requirements to maintain euglycemia after moderate-intensity afternoon exercise in adolescents with type 1 diabetes are increased in a biphasic manner. J. Clin. Endocrinol. Metab..

[56] Frier B.M. (2014). Hypoglycaemia in diabetes mellitus: Epidemiology and clinical implications. Nat. Rev. Endocrinol..

[57] Pedersen-Bjergaard U., Pramming S., Heller S.R., Wallace T.M., Rasmussen A.K., Jorgensen H.V., Matthews D.R., Hougaard P., Thorsteinsson B. (2004). Severe hypoglycaemia in 1076 adult patients with type 1 diabetes: Influence of risk markers and selection. Diabetes/Metab. Res. Rev..

[58] Sandoval D.A., Guy D.L.A., Richardson M.A., Ertl A.C., Davis S.N. (2006). Acute, same-day effects of antecedent exercise on counterregulatory responses to subsequent hypoglycemia in type 1 diabetes mellitus. Am. J. Physiol. Endocrinol. Metab..

[59] Cryer P.E. (2004). Diverse causes of hypoglycemia-associated autonomic failure in diabetes. N. Engl. J. Med..

[60] Booth G., Cheng A.Y. (2013). Canadian Diabetes Association 2013 clinical practice guidelines for the prevention and management of diabetes in Canada. Methods. Can. J. Diabetes.

[61] Hernandez J.M., Moccia T., Fluckey J.D., Ulbrecht J.S., Farrell P.A. (2000). Fluid snacks to help persons with type 1 diabetes avoid late onset postexercise hypoglycemia. Med. Sci. Sports Exerc..

[62] Yardley J.E., Kenny G.P., Perkins B.A., Riddell M.C., Balaa N., Malcolm J., Boulay P., Khandwala F., Sigal R.J. (2013). Resistance versus aerobic exercise: Acute effects on glycemia in type 1 diabetes. Diabetes Care.

[63] Yardley J.E., Kenny G.P., Perkins B.A., Riddell M.C., Malcolm J., Boulay P., Khandwala F., Sigal R.J. (2012). Effects of performing resistance exercise before versus after aerobic exercise on glycemia in type 1 diabetes. Diabetes Care.

[64] Turner D., Luzio S., Gray B.J., Bain S.C., Hanley S., Richards A., Rhydderch D.C., Martin R., Campbell M.D., Kilduff L.P. (2016). Algorithm that delivers an individualized rapid-acting insulin dose after morning resistance exercise counters post-exercise hyperglycaemia in people with Type 1 diabetes. Diabet. Med. A J. Br. Diabet. Assoc..

[65] Turner D., Gray B.J., Luzio S., Dunseath G., Bain S.C., Hanley S., Richards A., Rhydderch D.C., Ayles M., Kilduff L.P. (2016). Similar magnitude of post-exercise hyperglycemia despite manipulating resistance exercise intensity in type 1 diabetes individuals. Scand. J. Med. Sci. Sports.

[66] Eshghi S.R.T., Yardley J.E. (2017). Acute Effects of Morning versus Afternoon Resistance Exercise on Glycemia in Type 1 Diabetes. Can. J. Diabetes.

[67] Ruegemer J.J., Squires R.W., Marsh H.M., Haymond M.W., Cryer P.E., Rizza R.A., Miles J.M. (1990). Differences between prebreakfast and late afternoon glycemic responses to exercise in IDDM patients. Diabetes Care.

[68] Scott S.N., Cocks M., Andrews R.C., Narendran P., Purewal T.S., Cuthbertson D.J., Wagenmakers A.J.M., Shepherd S.O. (2019). Fasted High-Intensity Interval and Moderate-Intensity Exercise Do Not Lead to Detrimental 24-Hour Blood Glucose Profiles. J. Clin. Endocrinol. Metab..

[69] Iscoe K.E., Riddell M.C. (2011). Continuous moderate-intensity exercise with or without intermittent high-intensity work: Effects on acute and late glycaemia in athletes with Type 1 diabetes mellitus. Diabet. Med. A J. Br. Diabet. Assoc..

[70] Moser O., Tschakert G., Mueller A., Groeschl W., Pieber T.R., Obermayer-Pietsch B., Koehler G., Hofmann P. (2015). Effects of High-Intensity Interval Exercise versus Moderate Continuous Exercise on Glucose Homeostasis and Hormone Response in Patients with Type 1 Diabetes Mellitus Using Novel Ultra-Long-Acting Insulin. PLoS ONE.

[71] Guelfi K.J., Jones T.W., Fournier P.A. (2005). Intermittent high-intensity exercise does not increase the risk of early postexercise hypoglycemia in individuals with type 1 diabetes. Diabetes Care.

[72] Campbell M.D., West D.J., Bain S.C., Kingsley M.I., Foley P., Kilduff L., Turner D., Gray B., Stephens J.W., Bracken R.M. (2015). Simulated games activity vs continuous running exercise: A novel comparison of the glycemic and metabolic responses in T1DM patients. Scand. J. Med. Sci. Sports.

[73] Campbell P.J., Bolli G.B., Cryer P.E., Gerich J.E. (1985). Sequence of events during development of the dawn phenomenon in insulin-dependent diabetes mellitus. Metab. Clin. Exp..

[74] Edge J.A., Matthews D.R., Dunger D.B. (1990). The dawn phenomenon is related to overnight growth hormone release in adolescent diabetics. Clin. Endocrinol..

[75] Davidson M.B., Harris M.D., Ziel F.H., Rosenberg C.S. (1988). Suppression of sleep-induced growth hormone secretion by anticholinergic agent abolishes dawn phenomenon. Diabetes.

[76] Gonzalez J.T., Fuchs C.J., Betts J.A., van Loon L.J. (2017). Glucose Plus Fructose Ingestion for Post-Exercise Recovery-Greater than the Sum of Its Parts?. Nutrients.

[77] Mayes P.A. (1993). Intermediary metabolism of fructose. Am. J. Clin. Nutr..

[78] Smart C.E., Evans M., O’Connell S.M., McElduff P., Lopez P.E., Jones T.W., Davis E.A., King B.R. (2013). Both dietary protein and fat increase postprandial glucose excursions in children with type 1 diabetes, and the effect is additive. Diabetes Care.

[79] Paterson M.A., Smart C.E.M., Lopez P.E., Howley P., McElduff P., Attia J., Morbey C., King B.R. (2017). Increasing the protein quantity in a meal results in dose-dependent effects on postprandial glucose levels in individuals with Type 1 diabetes mellitus. Diabet. Med. A J. Br. Diabet. Assoc..

[80] Krebs J.D., Arahill J., Cresswell P., Weatherall M., Parry-Strong A. (2018). The effect of additional mealtime insulin bolus using an insulin-to-protein ratio compared to usual carbohydrate counting on postprandial glucose in those with type 1 diabetes who usually follow a carbohydrate-restricted diet: A randomized cross-over trial. Diabetes Obes. Metab..

[81] Bell K.J., Fio C.Z., Twigg S., Duke S.A., Fulcher G., Alexander K., McGill M., Wong J., Brand-Miller J., Steil G.M. (2019). Amount and Type of Dietary Fat, Postprandial Glycemia, and Insulin Requirements in Type 1 Diabetes: A Randomized Within-Subject Trial. Diabetes Care.

[82] Evans M., Smart C.E.M., Paramalingam N., Smith G.J., Jones T.W., King B.R., Davis E.A. (2019). Dietary protein affects both the dose and pattern of insulin delivery required to achieve postprandial euglycaemia in Type 1 diabetes: A randomized trial. Diabet. Med. A J. Br. Diabet. Assoc..

[83] Bell K.J., Smart C.E., Steil G.M., Brand-Miller J.C., King B., Wolpert H.A. (2015). Impact of fat, protein, and glycemic index on postprandial glucose control in type 1 diabetes: Implications for intensive diabetes management in the continuous glucose monitoring era. Diabetes Care.

[84] Bell K.J., Toschi E., Steil G.M., Wolpert H.A. (2016). Optimized Mealtime Insulin Dosing for Fat and Protein in Type 1 Diabetes: Application of a Model-Based Approach to Derive Insulin Doses for Open-Loop Diabetes Management. Diabetes Care.

[85] Neu A., Behret F., Braun R., Herrlich S., Liebrich F., Loesch-Binder M., Schneider A., Schweizer R. (2015). Higher glucose concentrations following protein- and fat-rich meals—The Tuebingen Grill Study: A pilot study in adolescents with type 1 diabetes. Pediatr. Diabetes.

[86] Association A.D. (2019). 5. Lifestyle Management: Standards of Medical Care in Diabetes—2019. Diabetes Care.

[87] Achten J., Jentjens R.L., Brouns F., Jeukendrup A.E. (2007). Exogenous oxidation of isomaltulose is lower than that of sucrose during exercise in men. J. Nutr..

[88] Nansel T.R., Gellar L., McGill A. (2008). Effect of varying glycemic index meals on blood glucose control assessed with continuous glucose monitoring in youth with type 1 diabetes on basal-bolus insulin regimens. Diabetes Care.

[89] Parillo M., Annuzzi G., Rivellese A.A., Bozzetto L., Alessandrini R., Riccardi G., Capaldo B. (2011). Effects of meals with different glycaemic index on postprandial blood glucose response in patients with Type 1 diabetes treated with continuous subcutaneous insulin infusion. Diabet. Med. A J. Br. Diabet. Assoc..

[90] Rovner A.J., Nansel T.R., Gellar L. (2009). The effect of a low-glycemic diet vs a standard diet on blood glucose levels and macronutrient intake in children with type 1 diabetes. J. Am. Diet. Assoc..

[91] West D.J., Morton R.D., Stephens J.W., Bain S.C., Kilduff L.P., Luzio S., Still R., Bracken R.M. (2011). Isomaltulose Improves Postexercise Glycemia by Reducing CHO Oxidation in T1DM. Med. Sci. Sports Exerc..

[92] Campbell M.D., Gonzalez J.T., Rumbold P.L., Walker M., Shaw J.A., Stevenson E.J., West D.J. (2015). Comparison of appetite responses to high- and low-glycemic index postexercise meals under matched insulinemia and fiber in type 1 diabetes. Am. J. Clin. Nutr..

[93] Campbell M.D., Walker M., Trenell M.I., Stevenson E.J., Turner D., Bracken R.M., Shaw J.A., West D.J. (2014). A low-glycemic index meal and bedtime snack prevents postprandial hyperglycemia and associated rises in inflammatory markers, providing protection from early but not late nocturnal hypoglycemia following evening exercise in type 1 diabetes. Diabetes Care.

[94] Bracken R.M., Page R., Gray B., Kilduff L.P., West D.J., Stephens J.W., Bain S.C. (2012). Isomaltulose improves glycemia and maintains run performance in type 1 diabetes. Med. Sci. Sports Exerc..

[95] Gray B.J., Page R., Turner D., West D.J., Campbell M.D., Kilduff L.P., Stephens J.W., Bain S.C., Bracken R.M. (2016). Improved end-stage high-intensity performance but similar glycemic responses after waxy barley starch ingestion compared to dextrose in type 1 diabetes. J. Sports Med. Phys. Fit..

[96] Kelley D., Mitrakou A., Marsh H., Schwenk F., Benn J., Sonnenberg G., Arcangeli M., Aoki T., Sorensen J., Berger M. (1988). Skeletal muscle glycolysis, oxidation, and storage of an oral glucose load. J. Clin. Investig..

[97] Ferrannini E., Bjorkman O., Reichard G.A., Pilo A., Olsson M., Wahren J., DeFronzo R.A. (1985). The disposal of an oral glucose load in healthy subjects. A quantitative study. Diabetes.

[98] Tappy L. (2018). Fructose metabolism and noncommunicable diseases: Recent findings and new research perspectives. Curr. Opin. Clin. Nutr. Metab. Care.

[99] Tappy L., Rosset R. (2017). Fructose Metabolism from a Functional Perspective: Implications for Athletes. Sports Med..

[100] Williams C.A. (1986). Metabolism of lactose and galactose in man. Prog. Biochem. Pharmacol..

[101] Bally L., Kempf P., Zueger T., Speck C., Pasi N., Ciller C., Feller K., Loher H., Rosset R., Wilhelm M. (2017). Metabolic Effects of Glucose-Fructose Co-Ingestion Compared to Glucose Alone during Exercise in Type 1 Diabetes. Nutrients.

[102] Kosinski C., Laesser C., Herzig D., Nakas C., Stettler C., Bally L. Pre-exercise intake of fructose reduces the risk of exercise-induced hypoglycaemia in individuals with type 1 diabetes on ultra-long acting insulin. Proceedings of the Schweizerischen Gesellschaft für Endokrinologie und Diabetologie (SGED).

[103] Ferraris R.P., Choe J.Y., Patel C.R. (2018). Intestinal Absorption of Fructose. Annu. Rev. Nutr..

[104] Lecoultre V., Benoit R., Carrel G., Schutz Y., Millet G.P., Tappy L., Schneiter P. (2010). Fructose and glucose co-ingestion during prolonged exercise increases lactate and glucose fluxes and oxidation compared with an equimolar intake of glucose. Am. J. Clin. Nutr..

[105] Gonzalez J.T., Fuchs C.J., Betts J.A., van Loon L.J. (2016). Liver glycogen metabolism during and after prolonged endurance-type exercise. Am. J. Physiol. Endocrinol. Metab..

[106] Jenni S., Oetliker C., Allemann S., Ith M., Tappy L., Wuerth S., Egger A., Boesch C., Schneiter P., Diem P. (2008). Fuel metabolism during exercise in euglycaemia and hyperglycaemia in patients with type 1 diabetes mellitus—A prospective single-blinded randomised crossover trial. Diabetologia.

[107] Beyer P.L., Caviar E.M., McCallum R.W. (2005). Fructose intake at current levels in the United States may cause gastrointestinal distress in normal adults. J. Am. Diet. Assoc..

[108] Lecoultre V., Egli L., Carrel G., Theytaz F., Kreis R., Schneiter P., Boss A., Zwygart K., Le K.A., Bortolotti M. (2013). Effects of fructose and glucose overfeeding on hepatic insulin sensitivity and intrahepatic lipids in healthy humans. Obesity.

[109] Faeh D., Minehira K., Schwarz J.M., Periasamy R., Park S., Tappy L. (2005). Effect of fructose overfeeding and fish oil administration on hepatic de novo lipogenesis and insulin sensitivity in healthy men. Diabetes.

[110] Egli L., Lecoultre V., Theytaz F., Campos V., Hodson L., Schneiter P., Mittendorfer B., Patterson B.W., Fielding B.A., Gerber P.A. (2013). Exercise prevents fructose-induced hypertriglyceridemia in healthy young subjects. Diabetes.

[111] Balakumar M., Raji L., Prabhu D., Sathishkumar C., Prabu P., Mohan V., Balasubramanyam M. (2016). High-fructose diet is as detrimental as high-fat diet in the induction of insulin resistance and diabetes mediated by hepatic/pancreatic endoplasmic reticulum (ER) stress. Mol. Cell. Biochem..

[112] Le K.A., Ith M., Kreis R., Faeh D., Bortolotti M., Tran C., Boesch C., Tappy L. (2009). Fructose overconsumption causes dyslipidemia and ectopic lipid deposition in healthy subjects with and without a family history of type 2 diabetes. Am. J. Clin. Nutr..

[113] Vlahek P., Car S., Ostroski I. (2013). Sweet 452 km—A report on the first type 1 diabetes patient to finish Double Ironman, a 30-hour endurance triathlon race. Croat. Med. J..

[114] van Dijk J.W., Eijsvogels T.M., Nyakayiru J., Schreuder T.H., Hopman M.T., Thijssen D.H., van Loon L.J. (2016). Glycemic control during consecutive days with prolonged walking exercise in individuals with type 1 diabetes mellitus. Diabetes Res. Clin. Pract..

[115] Murillo S., Brugnara L., Novials A. (2010). One year follow-up in a group of half-marathon runners with type-1 diabetes treated with insulin analogues. J. Sports Med. Phys. Fit..

[116] Gawrecki A., Zozulinska-Ziolkiewicz D., Matejko B., Hohendorff J., Malecki M.T., Klupa T. (2018). Safe Completion of a Trail Running Ultramarathon by Four Men with Type 1 Diabetes. Diabetes Technol. Ther..

[117] Hartvig Jensen T., Darre E., Holmich P., Jahnsen F. (1987). Insulin-dependent diabetes mellitus and marathon running. Br. J. Sports Med..

[118] Grimm J.J., Muchnick S. (1993). Type I diabetes and marathon running. Diabetes Care.

[119] Cauza E., Hanusch-Enserer U., Strasser B., Ludvik B., Kostner K., Dunky A., Haber P. (2005). Continuous glucose monitoring in diabetic long distance runners. Int. J. Sports Med..

[120] Graveling A.J., Frier B.M. (2010). Risks of marathon running and hypoglycaemia in Type 1 diabetes. Diabet. Med. A J. Br. Diabet. Assoc..

[121] Belli T., de Macedo D.V., Scariot P.P.M., de Araujo G.G., Dos Reis I.G.M., Lazarim F.L., Nunes L.A.S., Brenzikofer R., Gobatto C.A. (2017). Glycemic Control and Muscle Damage in 3 Athletes With Type 1 Diabetes During a Successful Performance in a Relay Ultramarathon: A Case Report. Wilderness Environ. Med..

[122] Bach C.W., Baur D.A., Hyder W.S., Ormsbee M.J. (2017). Blood glucose kinetics and physiological changes in a type 1 diabetic finisher of the Ultraman triathlon: A case study. Eur. J. Appl. Physiol..

[123] Khodaee M., Riederer M., VanBaak K., Hill J.C. (2015). Ultraendurance athletes with type 1 diabetes: Leadville 100 experience. Wilderness Environ. Med..

[124] Koivisto V.A., Sane T., Fyhrquist F., Pelkonen R. (1992). Fuel and Fluid Homeostasis During Long-Term Exercise in Healthy Subjects and Type I Diabetic Patients. Diabetes Care.

[125] Sane T., Helve E., Pelkonen R., Koivisto V.A. (1988). The adjustment of diet and insulin dose during long-term endurance exercise in type 1 (insulin-dependent) diabetic men. Diabetologia.

[126] Adolfsson P., Mattsson S., Jendle J. (2015). Evaluation of glucose control when a new strategy of increased carbohydrate supply is implemented during prolonged physical exercise in type 1 diabetes. Eur. J. Appl. Physiol..

[127] Mattsson S., Jendle J., Adolfsson P. (2019). Carbohydrate Loading Followed by High Carbohydrate Intake During Prolonged Physical Exercise and Its Impact on Glucose Control in Individuals With Diabetes Type 1-An Exploratory Study. Front. Endocrinol..

[128] Thomas D.T., Erdman K.A., Burke L.M. (2016). Position of the Academy of Nutrition and Dietetics, Dietitians of Canada, and the American College of Sports Medicine: Nutrition and Athletic Performance. J. Acad. Nutr. Diet..

[129] Burke L.M. (2001). Nutritional practices of male and female endurance cyclists. Sports Med..

[130] Jeukendrup A. (2014). A step towards personalized sports nutrition: Carbohydrate intake during exercise. Sports Med..

[131] Kime N.H., Pringle A., Rivett M.J., Robinson P.M. (2018). Physical activity and exercise in adults with type 1 diabetes: Understanding their needs using a person-centered approach. Health Educ. Res..

[132] Hearris M.A., Hammond K.M., Fell J.M., Morton J.P. (2018). Regulation of Muscle Glycogen Metabolism during Exercise: Implications for Endurance Performance and Training Adaptations. Nutrients.

[133] Elliott-Sale K.J., Tenforde A.S., Parziale A.L., Holtzman B., Ackerman K.E. (2018). Endocrine Effects of Relative Energy Deficiency in Sport. Int. J. Sport Nutr. Exerc. Metab..

[134] Mountjoy M., Sundgot-Borgen J., Burke L., Carter S., Constantini N., Lebrun C., Meyer N., Sherman R., Steffen K., Budgett R. (2014). The IOC consensus statement: Beyond the Female Athlete Triad—Relative Energy Deficiency in Sport (RED-S). Br. J. Sports Med..

[135] Wiesli P., Schmid C., Kerwer O., Nigg-Koch C., Klaghofer R., Seifert B., Spinas G.A., Schwegler K. (2005). Acute psychological stress affects glucose concentrations in patients with type 1 diabetes following food intake but not in the fasting state. Diabetes Care.

[136] Yardley J.E., Zaharieva D.P., Jarvis C., Riddell M.C. (2015). The “ups” and “downs” of a bike race in people with type 1 diabetes: Dramatic differences in strategies and blood glucose responses in the Paris-to-Ancaster Spring Classic. Can. J. Diabetes.

[137] Hawley J.A., Schabort E.J., Noakes T.D., Dennis S.C. (1997). Carbohydrate-loading and exercise performance. An update. Sports Med..

[138] McKewen M.W., Rehrer N.J., Cox C., Mann J. (1999). Glycaemic control, muscle glycogen and exercise performance in IDDM athletes on diets of varying carbohydrate content. Int. J. Sports Med..

[139] Hwang J.H., Perseghin G., Rothman D.L., Cline G.W., Magnusson I., Petersen K.F., Shulman G.I. (1995). Impaired net hepatic glycogen synthesis in insulin-dependent diabetic subjects during mixed meal ingestion. A 13C nuclear magnetic resonance spectroscopy study. J. Clin. Investig..

[140] Bischof M.G., Krssak M., Krebs M., Bernroider E., Stingl H., Waldhausl W., Roden M. (2001). Effects of short-term improvement of insulin treatment and glycemia on hepatic glycogen metabolism in type 1 diabetes. Diabetes.

[141] Edgerton D.S., Scott M., Farmer B., Williams P.E., Madsen P., Kjeldsen T., Brand C.L., Fledelius C., Nishimura E., Cherrington A.D. (2019). Targeting insulin to the liver corrects defects in glucose metabolism caused by peripheral insulin delivery. JCI Insight.

[142] Bally L., Buehler T., Dokumaci A.S., Boesch C., Stettler C. (2015). Hepatic and intramyocellular glycogen stores in adults with type 1 diabetes and healthy controls. Diabetes Res. Clin. Pract..

[143] Brockman N.K., Yardley J.E. (2018). Sex-related differences in fuel utilization and hormonal response to exercise: Implications for individuals with type 1 diabetes. Appl. Physiol. Nutr. Metab. Physiol. Appl. Nutr. Metab..

[144] Yardley J.E., Brockman N.K., Bracken R.M. (2018). Could Age, Sex and Physical Fitness Affect Blood Glucose Responses to Exercise in Type 1 Diabetes?. Front. Endocrinol..

[145] Devries M.C. (2016). Sex-based differences in endurance exercise muscle metabolism: Impact on exercise and nutritional strategies to optimize health and performance in women. Exp. Physiol..

[146] Tarnopolsky M.A., Ruby B.C. (2001). Sex differences in carbohydrate metabolism. Curr. Opin. Clin. Nutr. Metab. Care.

[147] Carter S.L., Rennie C., Tarnopolsky M.A. (2001). Substrate utilization during endurance exercise in men and women after endurance training. Am. J. Physiol. Endocrinol. Metab..

[148] Devries M.C., Hamadeh M.J., Phillips S.M., Tarnopolsky M.A. (2006). Menstrual cycle phase and sex influence muscle glycogen utilization and glucose turnover during moderate-intensity endurance exercise. Am. J. Physiol. Regul. Integr. Comp. Physiol..

[149] Romijn J.A., Coyle E.F., Sidossis L.S., Rosenblatt J., Wolfe R.R. (2000). Substrate metabolism during different exercise intensities in endurance-trained women. J. Appl. Physiol..

[150] Tarnopolsky L.J., MacDougall J.D., Atkinson S.A., Tarnopolsky M.A., Sutton J.R. (1990). Gender differences in substrate for endurance exercise. J. Appl. Physiol..

[151] Carter S., McKenzie S., Mourtzakis M., Mahoney D.J., Tarnopolsky M.A. (2001). Short-term 17beta-estradiol decreases glucose R(a) but not whole body metabolism during endurance exercise. J. Appl. Physiol..

[152] Riddell M.C., Partington S.L., Stupka N., Armstrong D., Rennie C., Tarnopolsky M.A. (2003). Substrate utilization during exercise performed with and without glucose ingestion in female and male endurance trained athletes. Int. J. Sport Nutr. Exerc. Metab..

[153] Mihm M., Gangooly S., Muttukrishna S. (2011). The normal menstrual cycle in women. Anim. Reprod. Sci..

[154] Brown S.A., Jiang B., McElwee-Malloy M., Wakeman C., Breton M.D. (2015). Fluctuations of Hyperglycemia and Insulin Sensitivity Are Linked to Menstrual Cycle Phases in Women With T1D. J. Diabetes Sci. Technol..

[155] Ruby B.C., Robergs R.A., Waters D.L., Burge M., Mermier C., Stolarczyk L. (1997). Effects of estradiol on substrate turnover during exercise in amenorrheic females. Med. Sci. Sports Exerc..

[156] Devries M.C., Hamadeh M.J., Graham T.E., Tarnopolsky M.A. (2005). 17beta-estradiol supplementation decreases glucose rate of appearance and disappearance with no effect on glycogen utilization during moderate intensity exercise in men. J. Clin. Endocrinol. Metab..

[157] D’Eon T.M., Sharoff C., Chipkin S.R., Grow D., Ruby B.C., Braun B. (2002). Regulation of exercise carbohydrate metabolism by estrogen and progesterone in women. Am. J. Physiol. Endocrinol. Metab..

[158] Zderic T.W., Coggan A.R., Ruby B.C. (2001). Glucose kinetics and substrate oxidation during exercise in the follicular and luteal phases. J. Appl. Physiol..

[159] Campbell S.E., Angus D.J., Febbraio M.A. (2001). Glucose kinetics and exercise performance during phases of the menstrual cycle: Effect of glucose ingestion. Am. J. Physiol. Endocrinol. Metab..

[160] Baechle C., Castillo K., Strassburger K., Stahl-Pehe A., Meissner T., Holl R.W., Giani G., Rosenbauer J. (2014). Is disordered eating behavior more prevalent in adolescents with early-onset type 1 diabetes than in their representative peers?. Int. J. Eat. Disord..

[161] Wisting L., Froisland D.H., Skrivarhaug T., Dahl-Jorgensen K., Ro O. (2013). Disturbed eating behavior and omission of insulin in adolescents receiving intensified insulin treatment: A nationwide population-based study. Diabetes Care.

[162] Roy T., Lloyd C.E. (2012). Epidemiology of depression and diabetes: A systematic review. J. Affect. Disord..

[163] Anderson R.J., Freedland K.E., Clouse R.E., Lustman P.J. (2001). The prevalence of comorbid depression in adults with diabetes: A meta-analysis. Diabetes Care.

[164] Cash T.F., Novy P.L., Grant J.R. (1994). Why do women exercise? Factor analysis and further validation of the Reasons for Exercise Inventory. Percept. Mot. Ski..

[165] Francescato M.P., Geat M., Fusi S., Stupar G., Noacco C., Cattin L. (2004). Carbohydrate requirement and insulin concentration during moderate exercise in type 1 diabetic patients. Metab. Clin. Exp..

[166] Neumark-Sztainer D., Patterson J., Mellin A., Ackard D.M., Utter J., Story M., Sockalosky J. (2002). Weight control practices and disordered eating behaviors among adolescent females and males with type 1 diabetes: Associations with sociodemographics, weight concerns, familial factors, and metabolic outcomes. Diabetes Care.

[167] Young V., Eiser C., Johnson B., Brierley S., Epton T., Elliott J., Heller S. (2013). Eating problems in adolescents with Type 1 diabetes: A systematic review with meta-analysis. Diabet. Med. A J. Br. Diabet. Assoc..

[168] Litchfield I., Andrews R.C., Narendran P., Greenfield S. (2019). Patient and Healthcare Professionals Perspectives on the Delivery of Exercise Education for Patients with Type 1 Diabetes. Front. Endocrinol..

[169] Francescato M.P., Stel G., Stenner E., Geat M. (2015). Prolonged exercise in type 1 diabetes: Performance of a customizable algorithm to estimate the carbohydrate supplements to minimize glycemic imbalances. PLoS ONE.

[170] Buoite Stella A., Assaloni R., Tonutti L., Manca E., Tortul C., Candido R., Francescato M.P. (2017). Strategies used by Patients with Type 1 Diabetes to Avoid Hypoglycemia in a 24x1-Hour Marathon: Comparison with the Amounts of Carbohydrates Estimated by a Customizable Algorithm. Can. J. Diabetes.

[171] Francescato M.P., Carrato S. (2011). Management of exercise-induced glycemic imbalances in type 1 diabetes. Curr. Diabetes Rev..

[172] Jacobs P.G., Resalat N., El Youssef J., Reddy R., Branigan D., Preiser N., Condon J., Castle J. (2015). Incorporating an Exercise Detection, Grading, and Hormone Dosing Algorithm Into the Artificial Pancreas Using Accelerometry and Heart Rate. J. Diabetes Sci. Technol..

